# RFPL3 and CBP synergistically upregulate hTERT activity and promote lung cancer growth

**DOI:** 10.18632/oncotarget.4825

**Published:** 2015-08-11

**Authors:** Yu Qin, Wangbing Chen, Yao Xiao, Wendan Yu, Xin Cai, Meng Dai, Tingting Xu, Wenlin Huang, Wei Guo, Wuguo Deng, Taihua Wu

**Affiliations:** ^1^ The First Affiliated Hospital and Institute of Cancer Stem Cell, Dalian Medical University, Dalian, China; ^2^ Sun Yat-Sen University Cancer Center, State Key Laboratory of Oncology in South China, Collaborative Innovation Center of Cancer Medicine, Guangzhou, China; ^3^ Cancer Center, Union Hospital, Tongji Medical College, Huazhong University of Science and Technology, Wuhan, China; ^4^ State Key Laboratory of Targeted Drug for Tumors of Guangdong Province, Guangzhou Double Bioproduct Inc., Guangzhou, China

**Keywords:** RFPL3, CBP, hTERT, lung cancer

## Abstract

hTERT is the key component of telomerase and its overactivation contributes to maintaining telomere length and cell immortalization. Previously, we identified RFPL3 as a new transcription activator of hTERT in lung cancers. However, the exact mechanism of RFPL3 in mediating hTERT activation and its associated signal regulatory network remain unclear. In this study, we found that RFPL3 colocalized and interacted directly with CBP in the nucleus of lung cancer cells. Immunohistochemical analysis of tissue microarrays of lung cancers revealed the simultaneous overexpression of both RFPL3 and CBP predicted relatively poor prognosis. Furthermore, we confirmed their synergistic stimulation on hTERT expression and tumor cell growth. The binding of RFPL3 to hTERT promoter was reduced markedly when CBP was knocked down by its specific siRNA or suppressed by its inhibitor in lung cancer cells with stable overexpression of RFPL3. When one of the two proteins RFPL3 and CBP was upregulated or downregulated, whereas the another remains unchanged, hTERT expression and telomerase activity were activated or repressed accordingly. In the meantime, the growth of lung cancer cells was also promoted or attenuated accordingly. Furthermore, we also found that RFPL3 coordinated with CBP to upregulate hTERT through the CBP-induced acetylation of RFPL3 protein and their co-anchoring at hTERT promoter region. Collectively, our results reveal a new mechanism of hTERT regulation in lung cancer cells and suggest the RFPL3/CBP/hTERT signaling pathway may be a new targets for lung cancer treatment.

## INTRODUCTION

Telomerase, a ribonucleoprotein complex located at the ends of linear chromosomes, is responsible for maintaining unlimited chromosome replication and continuous cell proliferation in cancer [1]. The observed correlation between telomerase activity and carcinogenesis has made it of great interest in cancer research. The human telomerase reverse transcriptase (hTERT) is the key component with regard to telomerase activity [2, 3]. In cells where telomerase is activated, hTERT synthesizes a TTAGGG sequence from the RNA template that is then added to the end of the shortening chromosome, thus saving the cells from aging or death [4, 5]. Considering its broad expression in 85% of all cancers, and yet little or no expression in normal somatic cells [6, 7], hTERT has been regarded as one hallmark of cancer and becomes a potential molecular target for cancer therapeutic interventions [8–10].

Telomerase activity can be elicited in many different ways [11–13], among which transcriptional regulation of hTERT gene is the limited step. A number of factors have been identified to regulate the activity of the hTERT promoter directly or indirectly, including cellular transcriptional activators (c-Myc, Sp1, HIF-1, AP-2, etc) [14–16] as well as the repressors such as CtBP, p53, WT1, etc [17–18]. However, little is known about the underlying molecular mechanisms of the reactivation of hTERT during tumorigenesis for lung cancers. In our previous study, we discovered and identified RFPL3 as a novel hTERT promoter-binding protein which could upregulate hTERT activity in lung cancers [19]. We also showed that the inhibition of RFPL3 expression significantly suppressed lung cancer cell growth *in vitro* and in a xenograft mouse model *in vivo*. Furthermore, the overexpression of RFPL3 was found to be significantly associated with lymph node metastasis of lung cancers and shorter OS of patients with lung adenocarcinomas. Also, its overexpression predicted poor prognosis in lung cancer patients. However, the exact molecular mechanism by which RFPL3 mediates hTERT activation and RFPL3-associated signal regulatory networks during carcinogenesis remains unclear.

CREB binding protein (CBP) is considered a cardinal transcriptional co-activator participating in a variety of intracellular processes under normal and pathologic conditions, which is similar to p300 [20, 21]. A variety of transcription factors that are regarded as oncoproteins, such as c-Fos, c-Jun, c-Myb, are downregulated by knockdown of CBP, which lead to a suppression of cell growth [22]. However, the high prevalence of malignant tumours among RTS patients whose CBP and p300 proteins are targets of transforming viruses, suggests that disruption of CBP function contributes to carcinogenesis [23]. Several studies have suggested its role as a co-activator through recruiting other transcriptional factors in promoting the expression of some key proteins involved in tumorigenesis and development [22, 24]. Intriguingly, it has been demonstrated that CBP was highly expressed in lung tumor cells and tumor tissues, and CBP upregulated hTERT expression and promoted tumor growth in human lung cancer cells [25]. CBP over-expression by increasing members of the activator protein-1 (AP-1) family and downregulating the retinoid acid receptor β might promote lung tumor progression and proliferation [26]. Furthermore, it has been found to exert its actions mainly via acetylating histones and other regulatory proteins [27, 28]. Based on the common transcriptional regulatory function of CBP, we proposed that it might be a helper of RFPL3 in mediating hTERT activation in lung carcinoma. In this study, we investigated their possible association and synergistic effect on the transcriptional regulation of hTERT and the growth of lung cancer cells and elucidated the underlying molecular mechanisms. Our study will reveal a new activated mechanism of hTERT regulation in lung cancer cells and explore the RFPL3/CBP/hTERT signaling as a potential target for lung cancer treatment.

## RESULTS

### RFPL3 and CBP is correlated with hTERT expression in lung cancer cell lines and tumor tissues

**Figure 1:**
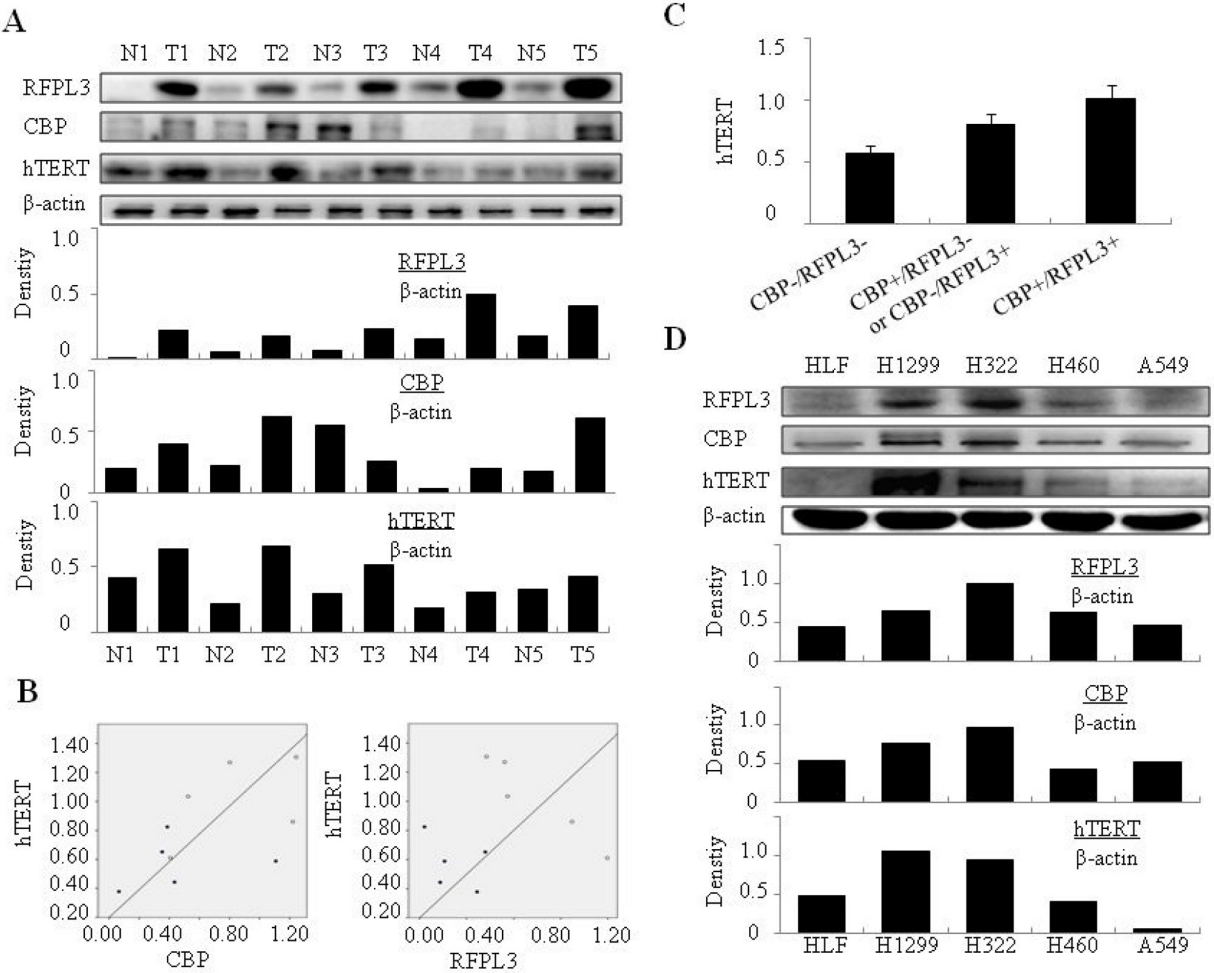
The positive correlation between RFPL3, CBP and hTERT expression in NSCLC cells and tissues **A.** NSCLC patients` fresh tumor specimens and matched normal lung tissues (distance from tumor over 3 cm) were gathered and the expressions of RFPL3, CBP and hTERT proteins were tested by Western blot. Densitometric analysis was used to quantitatively analyze the level of protein bands. **B.** RFPL3 and CBP protein expressions were respectively correlated positively with hTERT expression in tumor tissues through quantitative analysis of the Western blot data. (Pearson's Correlation test, *r* = 0.55; *P* < 0.001). **C.** The average expression level of hTERT in the tumor tissues with both high expression of RFPL3 and CBP, or with both low expression of RFPL3 and CBP, or with one high and the other low based on the quantitative analysis of the Western blot data. CBP-/RFPL3-: simultaneous low expression of RFPL3 and CBP, CBP+/RFPL3- or CBP-/RFPL3+: one high and the other low among RFPL3 and CBP, CBP+/RFPL3+: simultaneous high expression of RFPL3 and CBP. **D.** Expression of RFPL3, CBP, and hTERT in the NSCLC cell lines (H1299, H460, H322, A549) and in normal lung cell lines (HLF) by Western blot.

We first determined the expressions of RFPL3, CBP and hTERT in the fresh tumor specimens and their matched adjacent lung tissues (distance from tumor over 3 cm) from 5 cases of lung adenocarcinoma patients by Western blot analysis. It is obvious that RFPL3, CBP and hTERT proteins were upregulated in tumor tissues from most cases compared with the normal adjacent lung tissues (Figure 1A). Densitometric analysis was used to analyze quantitatively the level of these three proteins. The results showed that the expression of RFPL3 or CBP was positively correlated with hTERT protein respectively in lung tumor tissues (Figure 1B). We also analyzed the average expression levels of hTERT in the tumor tissues, and found that it was expressed more in the tumor tissues with high expression of both RFPL3 and CBP by comparison with those with lower expression of both RFPL3 and CBP, or with one high but the other low expression of RFPL3 and CBP (Figure 1C). In addition, Western blot and densitometric analysis showed that RFPL3, CBP and hTERT were overexpressed in lung cancer cell lines (H1299, H460, H322, A549) compared with the normal lung cell lines (HLF) (Figure 1D). These results therefore indicated the possible positive correlation among the expression of RFPL3, CBP and hTERT in lung cancer cells and the potential regulation of hTERT expression by CBP and RFPL3 in lung cancer.

### RFPL3 interacts with CBP in lung cancer cells

**Figure 2:**
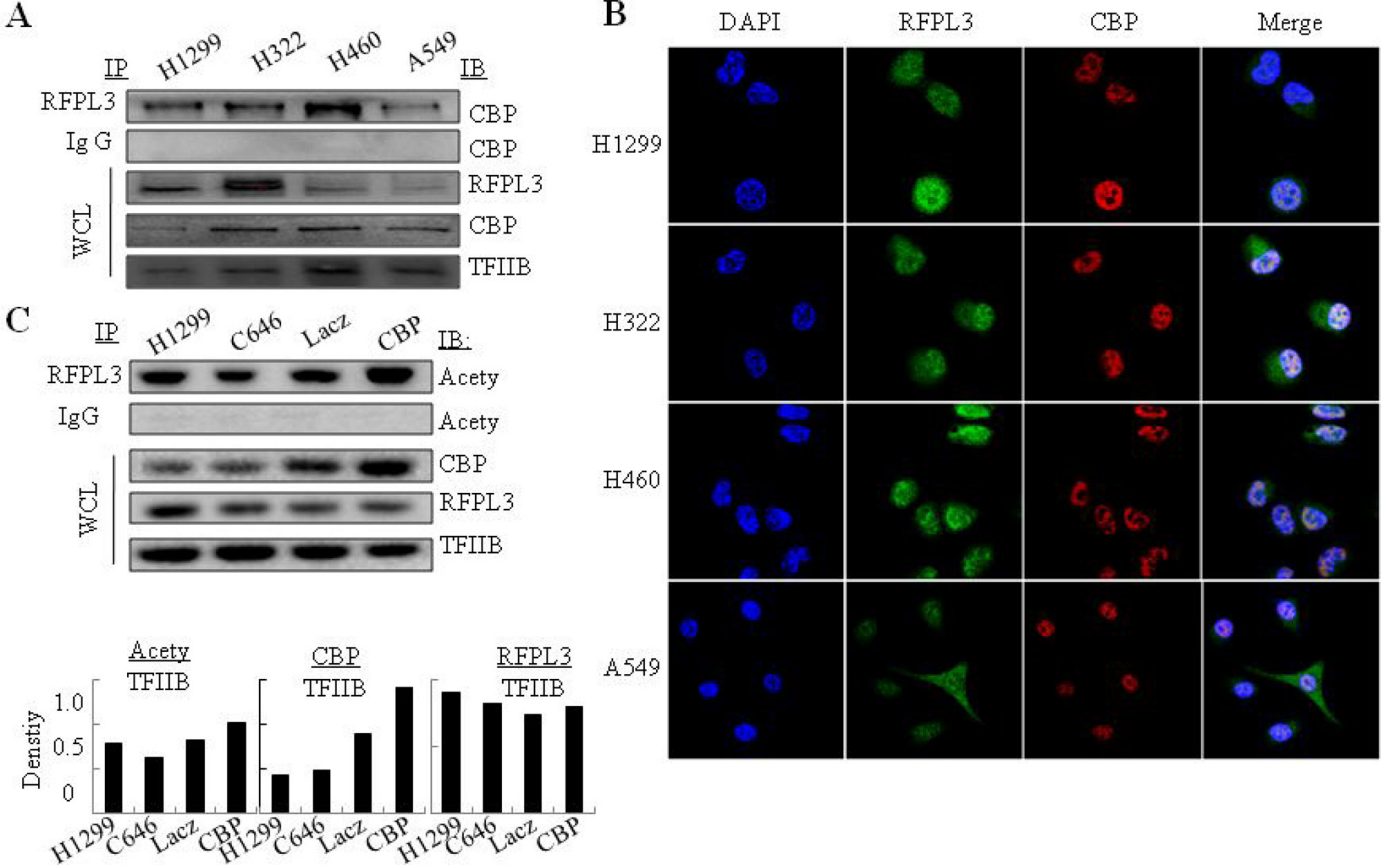
The interaction of RFPL3 with CBP and its acetylation by CBP in lung cancer cells **A.** The nuclear extracts of human lung cancer cells were prepared for immunoprecipitation using an antibody against RFPL3 and the immunoprecipitated complexes were then evaluated by immunoblot using antibody against CBP. IgG was used as negative control. The expression of RFPL3 and CBP in the nuclear extracts of H1299 cells were tested by Western blot analysis as WCL. **B.** Human lung cancer cells H1299, H322, H460 and A549 grown on chamber slides were cultivated for 24 h, and the subcellular localization and the colocalization of RFPL3 and CBP were examined by confocal microscopy analysis. Cells with typical morphology were presented. **C.** Immunoprecipitation was performed using antibody against RFPL3 in H1299 lung cancer cells respectively treated with Lac Z plasmids, or CBP plasmids, or CBP specific inhibitor (C646), and the acetylated RFPL3 was determined by immunoblot from immunoprecipitated complexes using the anti-acetylation antibody. IgG was used as negative control.

Since RFPL3 and CBP are overexpressed in lung cancer cells, a possible association between these two proteins might exist. We next used immunoprecipitation assay to determine their interaction. The nuclear extracts from lung cancer cell lines were immunoprecipitated using anti-RFPL3 antibody or the non-specific IgG control protein, respectively. The eluted proteins were evaluated by Western blot using antibody against CBP. The results showed that CBP was found in all the lung cancer cell lines in the complexes pulled down by anti-RFPL3 antibody, but not found in the IgG-treated samples (Figure 2A), indicating that RFPL3 indeed interacted directly with CBP in the nucleus of lung cancer cell lines. The RFPL3 and CBP expression in different lung cancer cell nucleus was also determined by Western blot assay (Figure 2A). To confirm the interaction between RFPL3 and CBP, dual immunofluorescence analysis was used to further analyze the co-localization of RFPL3 and CBP. Human lung cancer H1299, H322, H460 and A549 cells grown on chamber slides were cultivated for 24 hours, and the sub-cellular localization of RFPL3 and CBP and their co-localization were examined with a confocal microscope. The co-localization of RFPL3 and CBP in cell nuclei was detected in all four cell lines (Figure 2B). RFPL3 was also detected in the cytoplasm of cells, but distributed little.

### CBP mediates the acetylation of RFPL3 in lung cancer cells

CBP has been shown to function as a transcriptional coactivator through acetylating a variety of transcriptional factors. To determine whether CBP interacts with RFPL3 and acetylates the latter, immunoprecipitation was used to determine the levels of the acetylated RFPL3 in lung cancer cells. We incubated the nuclear extracts from different lung cancer cells with anti-RFPL3 antibody, and the acetylation level of RFPL3 was tested using an anti-acetylation antibody. As shown in Figure 2C, over-expression of CBP resulted in a significant increase in the acetylated level of RFPL3 compared with those transfected with LacZ plasmid. Conversely, inhibition of CBP activity by its specific inhibitor C646 attenuated the acetylation level of RFPL3 (Figure 2C), suggesting that CBP mediated the tumor-specific acetylation of RFPL3 and further interacted with each other in lung cancer cells.

Although CBP has been shown to up-regulate the expression of many cancer-related genes, it was interesting to discover that CBP could not regulate RFPL3 expression itself in human lung cancer cells from our results. The expression of RFPL3 protein kept nearly unchanged in lung cancer cells transfected with CBP plasmids (Figure 2C). The results demonstrated CBP-mediated acetylation of RFPL3 might be necessary pre-requisite for their interaction.

### The expression of RFPL3 and CBP is positively associated with hTERT and their expression predicates poor prognosis in lung cancer patients

**Figure 3:**
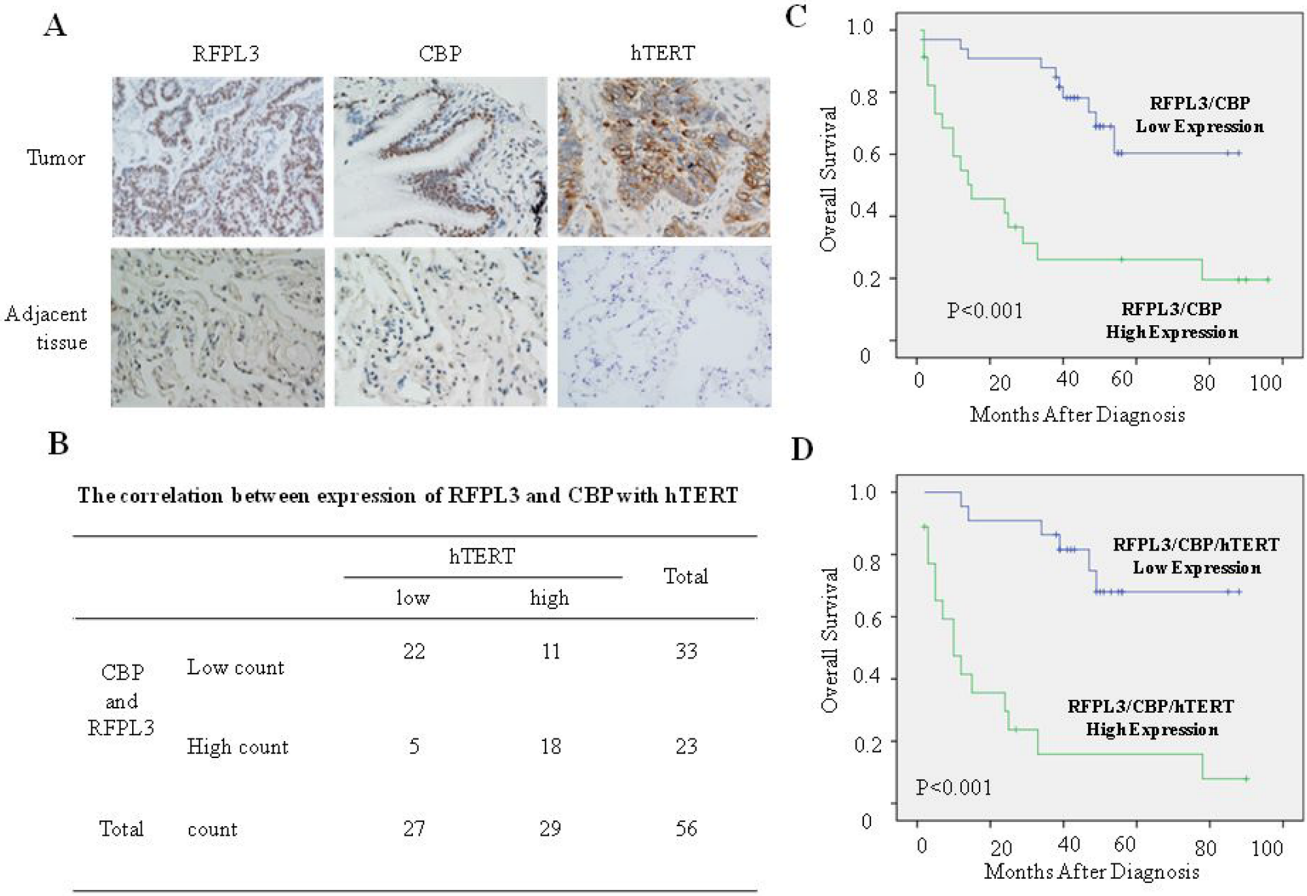
High expression of RFPL3, CBP and hTERT in lung adenocarcinomas tissues and their correlation with poor prognosis of patients with lung cancer **A.**The expression of RFPL3, CBP and hTERT protein in lung tumor tissues and corresponding adjacent normal lung tissues by immunohistochemistry analysis (magnification, × 200). If the final score was equal or bigger than four, the protein was considered high expression; otherwise, the protein was considered low expression. **B.** The relative quantitative analysis of hTERT protein expression in lung tumor tissues with both RFPL3 and CBP high expression or low expression. **C.** Kaplan–Meier analysis of overall survival of lung cancer patients with different RFPL3 and CBP expression (*p* < 0.001, log-rank test). **D.** Kaplan–Meier analysis of overall survival of lung cancer patients with different RFPL3, CBP and hTERT expression (*p* < 0.001, log-rank test).

The expression of RFPL3, CBP and hTERT protein was analyzed in lung tumor tissues from 100 cases of patients with lung adenocarcinoma by immunohistochemical assay based on tissue microarrays. The staining level of RFPL3, CBP and hTERT in the human lung adenocarcinoma specimens was scored by multiplying the intensity and the percentage value with a range from 0–12 and then the scores were analyzed. The protein was considered high expression with the score equal or bigger than four; otherwise, the protein was considered low expression. The potential positive correlation between the expression of RFPL3, CBP and hTERT in lung adenocarcinoma tissues were shown (Figure 3A). For the 100 lung adenocarcinoma cases, 23 cases showed high expression of RFPL3 and CBP, in which an average of 78% owned positive high hTERT staining. While only 33% showed high expression of hTERT in the remaining 33 cases with low expression of RFPL3 and CBP (*P* < 0.001, Figure 3B). Furthermore, the Pearson's correlation coefficient analysis showed that RFPL3 and CBP expression together were positively correlated with hTERT expression (*P* < 0.01).

The correlation between CBP and RFPL3 expression levels and clinicopathologic features of lung adenocarcinoma was further evaluated, and the results were summarized in Table 1A. The up-regulation of CBP and RFPL3 had no significantly association with patient's gender (*P* = 0.554, χ2 tests), age (*P* = 0.861, χ2 tests), *T* classification (*P* = 0.179, χ2 tests) and lymph node metastasis (*P* = 0.075, χ2 tests).

**Table 1A:** Association of RFPL3 and CBP expression with patient's clinicopathological features in lung ADC

	Total (*n* = 56)	RFPL3 and CBP low expression (*n* = 33)	RFPL3 and CBP high expression (*n* = 23)	*p*
Gender				
Male	27	17 (63%)	10 (37%)	0.554
Female	29	16 (55%)	13 (45%)	
Age,y				
<60	26	15	11	0.861
≥60	30	18	12	
pT factor				
T1+ T2	46	29	17	0.179
T3	10	4	6	
pN factor				
N0	39	26	13	0.075
N1+N2	17	7	10	

Abbreviations: ADC, adenocarcinoma

**Table 1B:** Cox proportional hazards model analysis of prognostic factors in patients with lung ADC

	HR	95% CI	Unfavorable/Favorable	*p*
Univariate analysis				
RFPL3 and CBP	4.353	1.947−9.733	High/low	<0.001^a^
Gender	0.754	0.422−1.348	Male/female	0.340
Age,y	0.997	0.559−1.778	≥60/<60	0.992
pT factor	1.962	1.015−3.793	T3/T1+T2	0.045^a^
pN factor	2.074	1.146−3.750	N1+N2/N0	0.016^a^
Multivariate analysis				
RFPL3 and CBP	4.052	1.774−9.259	High/low	0.001^a^
pT factor	0.848	0.329−2.156	T3/T1+T2	0.719
pN factor	1.388	0.604−3.192	N1+N2/N0	0.440

Abbreviations: ADC, adenocarcinoma; HR, hazard ratio; CI, confidence interval;

a *P* < 0.05

We further analyzed the synergistic effect of CBP, RFPL3 and hTERT expression on the survival rate of patients with lung adenocarcinomas by Kaplan–Meier analysis. Both high expression of CBP and RFPL3 predicted a shorter overall survival time in patients with lung adenocarcinomas compared with those with dual low expression of these two proteins (*P* < 0.001, log-rank test; Figure 3C). Moreover, the lung adenocarcinoma patients with simultaneously high expression of CBP, RFPL3 and hTERT had a significantly shorter OS than those with low CBP, RFPL3 and hTERT expression (*P* < 0.001, log-rank test; Figure 3D). Based on univariate analysis, the up-regulation of CBP and RFPL3 (*P* < 0.001), T3 stage (*P* = 0.045) and presence of lymph node metastasis (*P*= 0.016) were significant inferior prognostic factors for OS in patients with lung adenocarcinoma (Table 1B). Multivariate analysis further indicated that the up-regulation of CBP and RFPL3 (*P* = 0.001) was independent prognostic predictors for OS in patients with lung adenocarcinoma enrolled in this study (Table 1B), suggesting that the important role of CBP and RFPL3 expression and their association with hTERT in predicting the prognosis of patients with lung cancers.

### CBP knockdown or activity inhibition attenuates the RFPL3's binding at hTERT promoter and hTERT expression in H1299 cells mediated by RFPL3 overexpression

**Figure 4:**
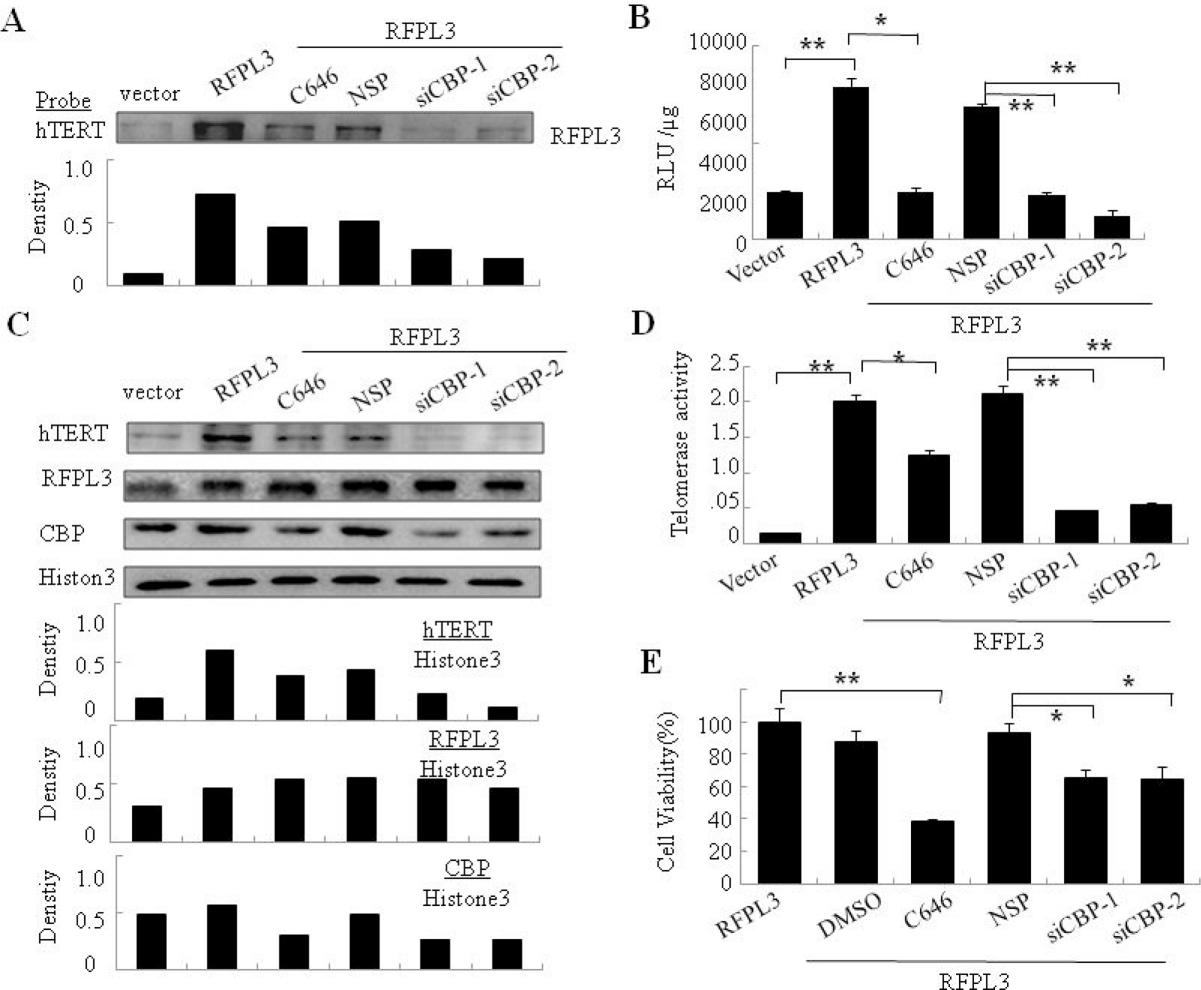
The synergistic regulation of hTERT promoter activity, hTERT expression, telomerase activity and cell proliferation in H1299 cells with overexpression of RFPL3 and low expression of CBP **A.** Streptavidin-agarose bead pulldown assay with hTERT promoter (−378 to +60) as probes was done in lung cancer cells (H1299 cells stably expressing RFPL3) treated by non-specifc siRNA or CBP specifc siRNA or CBP-specifc inhibitor(C646). The level of RFPL3 in the pulled down proteins was determined by immunoblot (NSP: non-specifc control siRNA). **B.** H1299 cells with stable overexpression of RFPL3 were co-transfected with CBP-specific siRNA1, or CBP-specific siRNA2 or CBP inhibitor and the plasmids of hTERT promoter driven-luciferase for 48 hours followed by luciferace assays. The relative luciferase intensity per μg protein was calculated in the treated cells. **C.** H1299 cells with stable overexpression of RFPL3 were transfected with CBP siRNA or incubated with CBP-specific inhibitor for 48 hours, and the expressions of hTERT, RFPL3 and CBP proteins in the nuclear extracts of these cells were examined by western blot. **D.** The telomerase activity in the cells treated as above was measured. **E.** Cell viability of these cells treated as above was measured by MTT assay. All of the measurements represent the means ± SE of three independent experiments. *P < 0.05, **P < 0.01, significant differences between treatment groups and control groups.

Since RFPL3 has been shown to be an hTERT promoter binding protein and it interacts with CBP in lung cancer cells [18], we next analyzed the effect of CBP on the RFPL3-mediated hTERT transcriptional activation. Knockdown of CBP by its-specific siRNA in H1299 cells with stable overexpression of RFPL3 markedly attenuated the binding of RFPL3 on hTERT promoter compared with those treated with non-specific siRNA (Figure 4A). In addition, the binding of RFPL3 to hTERT promoter was also reduced remarkably by the treatment with CBP inhibitor (Figure 4A). These results demonstrated that CBP might serve as a transcriptional coactivator through its co-accumulation with RFPL3 on the hTERT promoter to regulate hTERT expression.

To reveal whether CBP functions as a transcriptional coactivator to co-regulate hTERT promoter activity and expression with RFPL3, H1299 cells with RFPL3-stable overexpression were co-transfected with CBP-specific siRNA or treated with CBP inhibitor and hTERT promoter driven-luciferase plasmids. At 48 hours later, the expression of luciferase was assayed. Knockdown of CBP expression or inhibition of its activity significantly suppressed the expression of hTERT promoter-driven luciferase (Figure 4B). Similarly, Knockdown of CBP expression or inhibition of its activity significantly suppressed the expression of hTERT protein (Figure 4C).

### CBP knockdown or activity inhibition attenuates the up-regulated telomerase activity and cell growth in H1299 cells mediated by RFPL3 overexpression

We next investigated the effect of CBP on RFPL3-mediated telomerase activity in lung cancer cells. The H1299 cell lines stably expressing RFPL3 were transfected with CBP-specific siRNA or treated with CBP inhibitor. At 48 hours after treatment, the activity of telomerase were observed. As shown in Figure 4D, the telomerase activity was significantly attenuated by CBP knockdown or its activity inhibition, compared to the control groups.

Since hTERT was approved to be involved in the growth of lung cancer cells [19], and RFPL3 and CBP had been shown to be able to up-regulate hTERT expression, we next tested the coordinated effect of RFPL3 and CBP on cell proliferation in lung cancer cells. The H1299 cells with RFPL3-stable overexpression were transfected with CBP-specific siRNA or treated with CBP inhibitor C464. At 48 hours after treatment, the cell viability was assayed. Knockdown of CBP expression or inhibition of its activity significantly inhibited cell growth, compared with the cells themselves with stable overexpression of RFPL3 or the cells treated with the control siRNA (Figure 4E).

### RFPL3 knockdown reverses the up-regulated hTERT promoter activity, hTERT expression and cell growth in H1299 cells mediated by CBP overexpression

**Figure 5:**
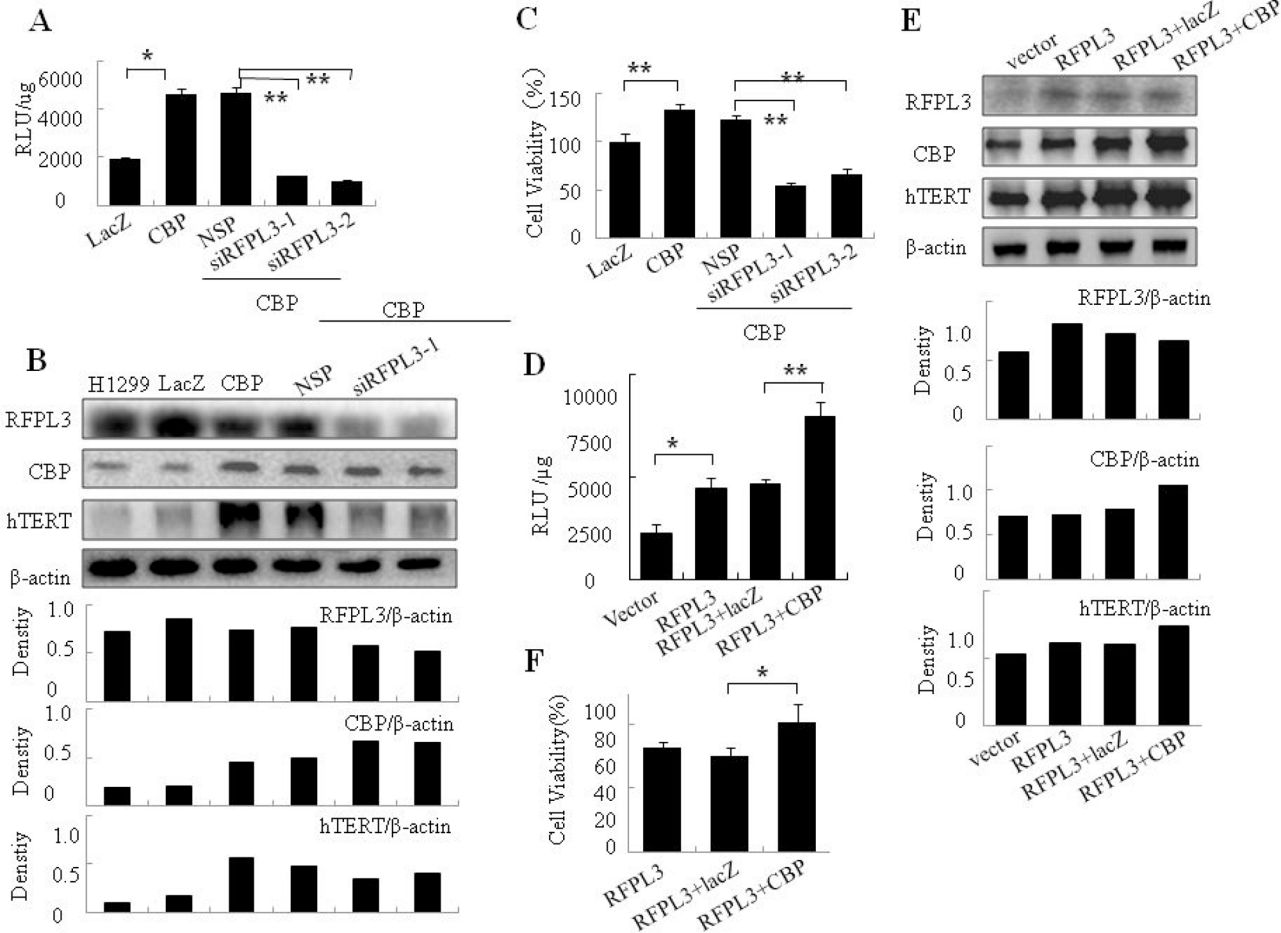
The co-regulation of hTERT promoter activity, hTERT expression, and cell proliferation in H1299 cells with overexpression of CBP and low expression of RFPL3 or simultaneous overexpression of CBP and RFPL3 **A.** H1299 cells were co-transfected with CBP plasmid or Lac Z plasmid, and RFPL3-specific siRNA (siRFPL3-1 and siRFPL3-2) or non-specific siRNA (NSP) and the plasmids of hTERT promoter driven-luciferase. 48 hours later, the relative luciferase intensity per μg protein was calculated in the treated cells. **B.** H1299 cells were co-transfected with CBP plasmid and RFPL3-specific siRNAs (siRFPL3-1 and siRFPL3-2) or non-specific siRNA (NSP). 48 hours after treatment, the expressions of hTERT, RFPL3 and CBP proteins in the lung cancer cell extracts were examined by western blot. **C.** Cell viability of these cells treated as above was assayed through MTT assay. **D.** H1299 cells stably overexpressing RFPL3 were co-transfected with CBP plasmid or Lac Z plasmid and the plasmids of hTERT promoter driven-luciferase. 48 hours later, the relative luciferase intensity per μg protein was calculated. **E.** H1299 cells with stable overexpression of RFPL3 were co-transfected with CBP plasmid or Lac Z plasmid. 48 hours after treatment, the expressions of hTERT, RFPL3 and CBP proteins in the lung cancer cells were examined by western blot. **F.** Cell viability was measured by MTT assay in H1299 cells with both overexpression of CBP and RFPL3 simultaneously.

In order to further confirm the coordinative regulation of hTERT transcription and expression by CBP and RFPL3, we used CBP-expressing plasmids, RFPL3-specific siRNA and hTERT promoter driven-luciferase plasmid to co-transfect H1299 cells. As shown in Figure 5A, overexpression of CBP up-regulated the activity of hTERT promoter driven-luciferase. However, knockdown of RFPL3 reversed the elevated activity of luciferase mediated by CBP overexpression. Accordingly, knockdown of RFPL3 in H1299 cells with CBP overexpression repressed remarkably the elevated expression of hTERT protein (Figure 5B) and lung cancer cell viability (Figure 5C) mediated by increased CBP expression, suggesting again the coordination between RFPL3 and CBP in mediating hTERT transcription and expression and their further role in regulating lung cancer cell growth.

### The co-regulation of hTERT promoter activity, hTERT expression, and cell proliferation in H1299 cells with simultaneous overexpression of CBP and RFPL3

Furthermore, we observed the effect of simultaneous overexpression of CBP and RFPL3 on hTERT transcription and expression. We transfected H1299 cells with stable overexpression of RFPL3 with CBP plasmids. 48 hours later, compared with the group treated with control vector Lac Z plasmids, the transcriptional activity of hTERT promoter was enhanced significantly (Figure 5D). In addition, the simultaneous overexpression of CBP and RFPL3, by means of transfecting CBP plasmids into H1299 cells with stable expression of RFPL3, further increased the elevated expression of hTERT protein mediated by RFPL3 overexpression (Figure 5E). Additionally, the simultaneous overexpression of CBP and RFPL3 promoted lung cancer cell growth obviously in H1299 cells by comparison to the group with stable overexpression of RFPL3 alone (Figure 5F), revealing again the potential cooperation between RFPL3 and CBP expression in regulating lung cancer cell survival.

### The effect of CBP on the binding of RFPL3 to hTERT promoter and their synergistic regulation on hTERT promoter activity and hTERT expression in H460 cells

**Figure 6:**
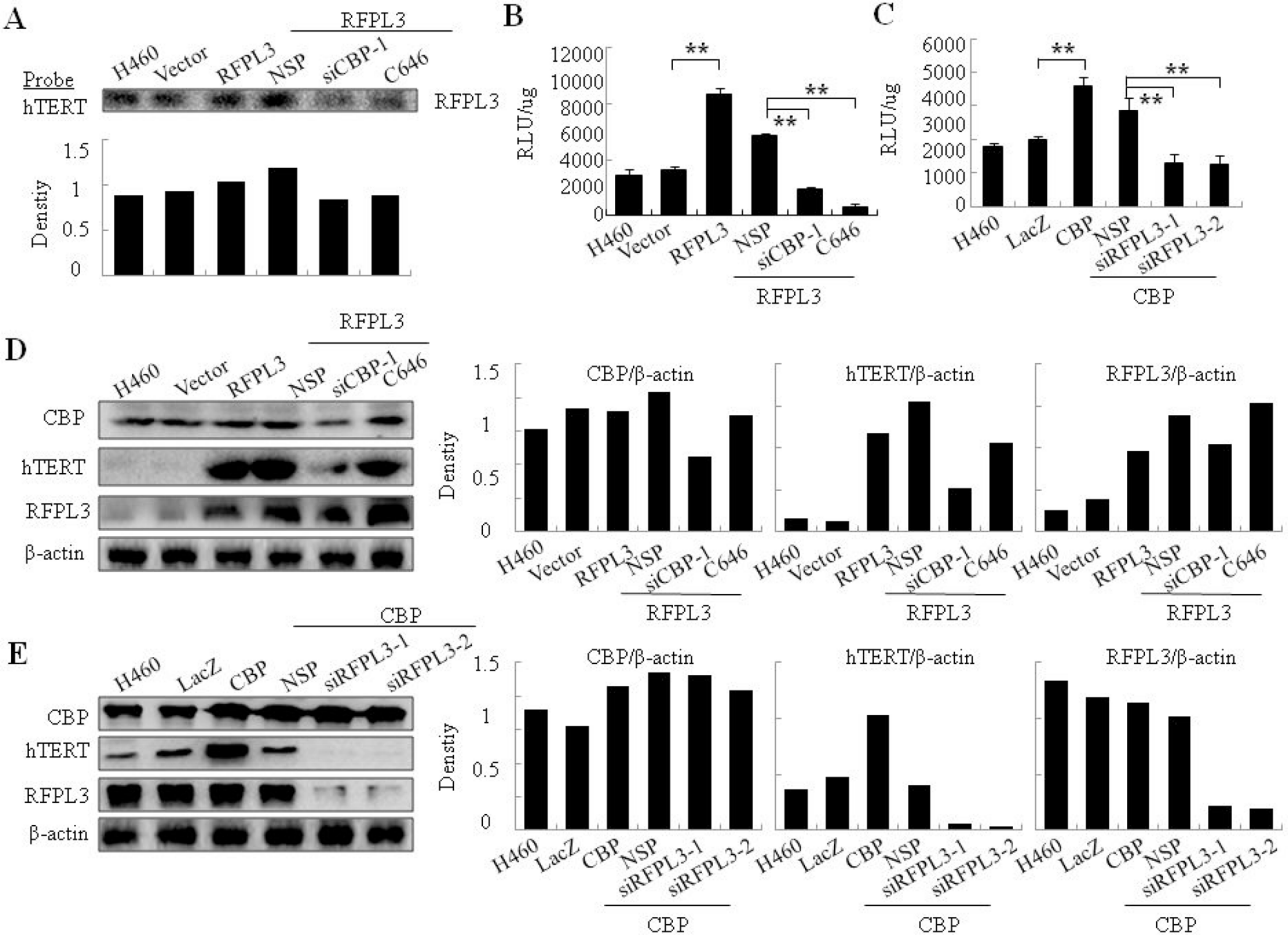
The synergistic regulation of hTERT promoter activity and hTERT expression in H460 cells by CBP and RFPL3 **A.** H460 cells were co-transfected with RFPL3 plasmid and CBP-specific siRNA1, or CBP inhibitor. The level of RFPL3 in the proteins pulled down by hTERT promoter probes was determined by immunoblot. **B.** H460 cells co-transfected with RFPL3 plasmids and CBP-specific siRNA1, or CBP inhibitor were co-incubated with the plasmids of hTERT promoter driven-luciferase for 48 hours. The luciferace activity was measured. **C.** H460 cells were co-transfected with CBP plasmids and RFPL3-specific siRNA and the plasmids of hTERT promoter driven-luciferase for 48 hours followed by luciferace activity assays. **D.** H460 cells were co-transfected with RFPL3 plasmid and CBP-specific siRNA1, or CBP inhibitor. The expressions of hTERT, RFPL3 and CBP proteins were examined by western blot. **E.** H460 cells were co-transfected with CBP plasmids and RFPL3-specific siRNAs. The expressions of hTERT, RFPL3 and CBP proteins were examined by western blot.

The co-regulation of hTERT transcription and expression by RFPL3 and CBP was also examined in H460 cells. The H460 cells were transfected with RFPL3 plasmids, or control LacZ plasmids, or co-treated with RFPL3 plasmids and CBP-specific siRNA, or RFPL3 plasmids and non-specific control siRNA, or RFPL3 plasmids and CBP inhibitor. 48 hours later, the binding of RFPL3 to hTERT promoter was determined by pulldown assay. The overexpression of RFPL3 elevated its binding to hTERT promoter, and such binding was remarkably reduced by knockdown of CBP or the treatment with CBP inhibitor (Figure 6A).

We next detected the synergistic regulation on hTERT promoter activity by RFPL3 and CBP in H460 cells. As shown in Figure 6B and 6C, CBP knockdown or its activity inhibition reversed the increased hTERT promoter activity caused by elevated RFPL3 expression. Similarly, RFPL3 knockdown reversed the elevated hTERT promoter activity mediated by over-expressed CBP.

Furthermore, the co-regulation of hTERT protein expression by RFPL3 and CBP was observed in H460 cells. Similarly, CBP knockdown or its activity inhibition reversed the increased hTERT protein expression caused by elevated RFPL3 expression, and nearly showed no effects on the expression of RFPL3 itself (Figure 6D). By contrast, RFPL3 knockdown reversed the increased hTERT protein expression caused by elevated CBP expression, and nearly caused no effects on the expression of CBP itself (Figure 6E).

## DISCUSSION

Overactivation of hTERT leads to telomere lengthening and cell immortalization, which plays a crucial role in tumorigenesis and development. Conversely, degradation of hTERT by proteasomes or repression of its activity prompts telomere shortening, cancer cell senescence and apoptosis [29] [30]. Therefore, hTERT has become an attractive target for anti-cancer therapy.

RFPL gene family first identified and cloned in 1999 [31] was comprised of three very similar genes, RFPL 1, 2 and 3, and the proteins encoded by them are members of a large protein family with zinc finger motifs [32, 33]. Some studies have revealed hRFPL1, 2, 3 play a vital role in neurogenesis [34, 35]. RFPL1 may participate in cell differentiation and cell-cycle regulation [36]. RFPL3 might enhance significantly the activity of human immunodeficiency virus, type 1 (HIV-1) PIC integration in infected cells *in vitro* [37]. However, the molecular expression and function of RFPL protein family, including RFPL3, in cancers are still unclear.

RFPL3 gene locates at human 22q12.3, which is much more closer to telomeres [31]. This gene locus facilitates its close relationship with hTERT. RFPL3 protein is usually detected as abundant transcripts in testis, adult brain, fetal brain, lung, as well as adrenal gland. Our results showed its upregulated expression in lung cancer and its role in regulating hTERT transcription. Nevertheless, RFPL3 protein lacks DNA-binding domain which is frequently essential for its binding to target gene promoter. Moreover, its special structural characteristics, such as RING-finger, coiled–coil, and B30–2 domains, which can easily mediate the protein-protein interaction by promoting homo- or heterodimerization [38–40]challenged us to guess the recruitment of the other transcriptional factors by it to the hTERT promoter elements.

Our previous research reported the overexpression of CBP in the lung cancer cells and tissues from patients with lung cancer [25]. In this study, we showed that the expression of RFPL3 and CBP was positively associated with hTERT expression and the approved function of CBP as transcriptional co-activator. We thus speculated that RFPL3 may cooperate with CBP to regulate hTERT transcription and telomerase activity in lung cancer. We verified that RFPL3 indeed interacted with CBP by immunoprecipitation analysis. Consistency of distribution is a prerequisite for the interaction, and duel-luciferase assay gives us another evidence that RFPL3 and CBP are colocalized in the nucleus of lung cancer cells. Furthermore, we confirmed their synergistic effect in mediating hTERT expression. When one of them was upregulated or downregulated, whereas the other one remains unchanged, hTERT expression and telomerase activity were activated or repressed accordingly. Therefore, all the results confirmed our hypothesis that RFPL3 may cooperate with CBP in the mediation of hTERT transcription and the molecular regulatory mechanisms for hTERT activation have been partially identified.

As multi-functional transcriptional co-activators, CBP binds concurrently to various proteins and act as a “bridge” between diverse transcription regulation proteins and co-factors [22]. Accordingly, CBP contributes to stabilize the transcription complex and increase the relative concentration of these transcriptional factors in the local environment. It has been noted that CBP participated in the regulation of COX-2 through co-anchoring with Ku80 at COX-2 promoter region of lung cancer cell lines [41]. CBP also has been reported to be cooperated with SP-1 and AP-2 to co-regulate hTERT expression in lung cancers [25]. Besides playing its transcriptional regulatory role through mediating the recruitment of basal transcriptional machinery to the promoter by interacting with other transcriptional factors, CBP also took part in transcription initiation by acetylating histones and other transcription proteins [42]. Hypo-acetylation generally correlates with transcriptional repression, and hyper-acetylation correlates with transcriptional activation [43, 44]. In this study, we also demonstrated the acetylation of RFPL3 mediated by CBP. Overexpression of CBP elevated the levels of RFPL3 acetylation, and conversely, the inhibition of its acetyltransferase activity reversed this elevation. Moreover, the changed expression level or HAT activity of CBP affected the binding of RFPL3 to hTERT promoter region. However, it caused no effect on the expression of RFPL3 itself. Accordingly, the expression level of RFPL3 caused no effect on the expression of CBP itself. Combined with some other studies, which have shown that acetylation of transcription factors enhanced their recognition by DNA and their DNA-binding activity, such as p53, p73, and E2F [45–47], our study indicated not only the acetylation of RFPL3 by CBP, but also the necessity of such acetylation for their co-anchoring at hTERT promoter region and further regulating hTERT transcription.

**Figure 7:**
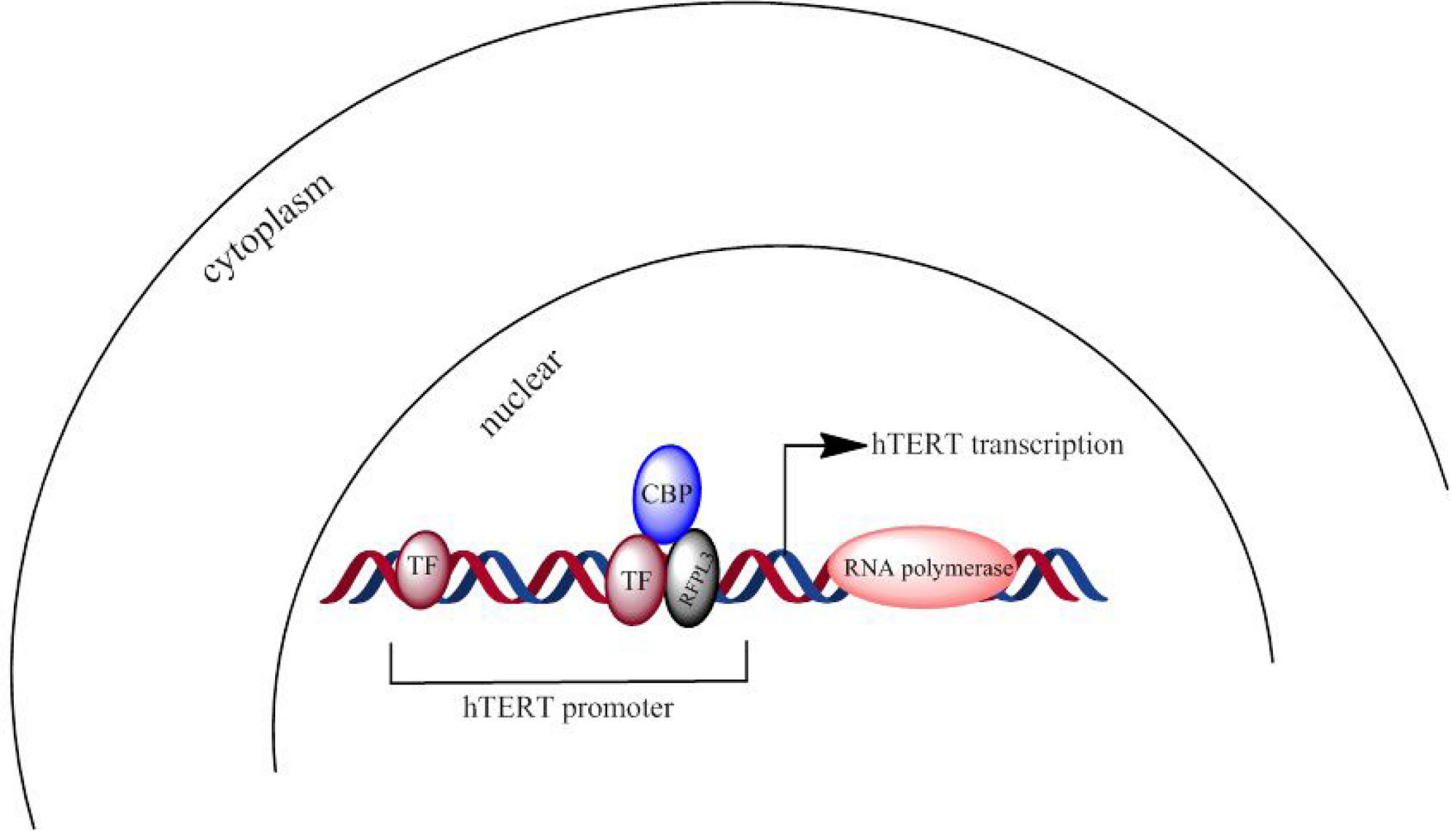
A scheme about RFPL3 and CBP's co-anchoring at hTERT promoter region and synergistic regulation on hTERT transcription (TF: transcriptional factor)

In summary, we revealed that RFPL3 coordinated with CBP to regulate hTERT promoter activity via the acetylation of RFPL3 by CBP and their co-anchoring at hTERT promoter region (Figure 7). Furthermore, we found their coordinative association and interdependence in promoting tumor cell proliferation. The simultaneous overexpression of RFPL3, CBP and hTERT predicted relatively poor prognosis of lung cancer patients, suggesting the most potential significance to inhibit lung cancer cell growth by downregulating the RFPL3/CBP/hTERT signaling pathway. Although the signal transduction mechanisms triggered by RFPL3 and its regulation on hTERT promoter activity are not entirely understood and further study is required, our results reveal a new mechanism of hTERT regulation in lung cancer cells and suggest the RFPL3/CBP/hTERT signaling pathway may be a new targets for lung cancer treatment.

## MATERIALS AND METHODS

### Cell lines and cell culture

Human lung cancer cell lines (H1299, H460, H322, A549), and human normal lung fibroblast (HLF), were obtained from the American Type Culture Collection (ATCC, Manassas, VA). HLF, H1299 and H322 were grown in the Dulbecco's modified Eagle's medium, DMEM (Invitrogen, Carlsbad, CA) supplemented with 10% fetal bovine serum. H460 and A549 were maintained in RPMI 1640 (Invitrogen, Carlsbad, CA) supplemented with 10% fetal bovine serum. All the cells were grown at 37°C in an atmosphere of 5% CO_2_. The lentivirus particles and plasmid for RFPL3 overexpression were designed and synthesized by Cyagen (Cyagen Biosciences Inc., United States). H1299 was used to generate stable cell lines in this study. H1299 cells with stable expression of RFPL3 were cultured in DMEM supplemented with 10% fetal bovine serum, 100 units/ml penicillin, and 100 units/ml streptomycin (Invitrogen, Carlsbad, CA).

### Western blot analysis

Proteins from cell lysate and tissue lysate were separated by electrophoresis in a 10% SDS-PAGE gels and electrophoretically transferred to a Hybond ECL nitrocellulose membrane (Amersham Pharmacia Biotech, Piscataway, NJ). Western blots probed with antibodies against hTERT (Millipore USA), β-Actin (Cell Signaling Technology, Beverly, MA), RFPL3 (Abcam), CBP, anti-acetylation antibody, histone 3 (Santa Cruz, USA) and TFIIB (Millipore, USA) were performed. The protein bands were visualized by enhanced chemiluminescence (Amersham Pharmacia Biotech, Piscataway, NJ) according to the manufacturer's instruction.

### Carcinoma Specimens

Lung cancer tissue specimens were collected for western blot analysis from patients with non small cell lung cancer (*n* = 5) and adjacent healthy tissues (*n* = 5). These patients were diagnosed with lung carcinoma and accepted surgery operation at the first affiliated hospital of Dalian Medical University (Dalian, China). Samples were stored at −80°C until analysis. Informed consent was obtained from each patient. This study was approved by the Committees on Human Rights in Research at Dalian Medical University.

### Nuclear extraction

Nuclear extraction was performed as previously described. Cells were harvested in 250 ul cytoplasm lysis buffer(10 mM Hepes, pH 7.9, 10 mM KCl, 1.5 mM MgCl_2_.6H_2_O, 0.5% NP-40, 300 mM Sucrose) with multiple protease inhibitors(1 mM Na_3_VO_4_, 10 mM NaF, 2.5 mM β-glycerophosphate, 0.1 mM PMSF, 1 g/ml leupeptin, and 0.5 mM dithiothreitol) and incubated on ice for 10 min. Vortexed briefly, and centrifuged at 2600 × g for 1 min at 4°C, the supernatant was transfered to a new tube, and stored at −80°C. The pellet was resuspended with 70–100 ul Nuclei lysis buffer(20 mM Hepes, pH 7.9, 420 mM NaCl, 1.5 mM MgCl_2_.6H_2_O, 0.1 mM EDTA, 2.5% Glycerol) with multiple protease inhibitors and kept on ice for 30–60 min. Nuclei proteins were extracted by centrifugation at 10400 × g for 10 min at 4°C. The supernatant was collected as nuclei proteins. Protein concentration was determined by BCA assay.

### Tissue protein extraction

The tissues were cut up in the pre-cool 0.9% NaCl, and transferred to the homogenate dish. The pre-cool lysis buffer 2 ml with protease inhibitors(Tris 50 mM, NaCl 150 mM, EDTA 0.5 mM, DTT 1mM, Triton X-100 1%, Deoxycholic acid sodium 0.5%, SDS 0.1%) was put into homogenate dish and the tissues were fully grinded in ice water. After centrifugation at 4°C, 14000 rpm for 10 min, the supernatant was transfered to new tube, and stored at −80°C

### Co-immunoprecipitation assays

Equal amounts of nuclear protein extracts prepared from different cell lines were incubated with the specific rabbit polyclonal antibodies against RFPL3, CBP or a non-immune rabbit IgG for overnight at 4°C. Then, the agarose-conjugated protein-A/G beads (Santa Cruz Biotech) were added into the immunocomplex and the mixture was incubated at 4°C for another 12 h. After extensive washing with ice-cold phosphate-buffered saline (PBS) with protease inhibitors, the beads were mixed with loading buffer and boiled. The proteins in the supernatant were separated by SDS-PAGE and transferred to NC membranes for Western blotting analysis.

### Plasmid vectors

Recombinant plasmid vectors pGL3-hTERT-438 expressing luciferase driven by a hTERT promoter (−378 to +60) were constructed in our lab and used in the transfection experiments. The CBP-expressing vector, pcDNA3.1-CBP or control vector pcDNA3.1-LacZ plasmids were designed and synthesized by Cyagen (Cyagen Biosciences Inc, United States). RFPL3 plasmids or control vector were designed and synthesized by Cyagen (Cyagen Biosciences Inc., United States).

### siRNA

The siRNAs targeting RFPL3 were purchased from ShangHai GenePharma Co (Shanghai, China). The sequence of siRNA1–565 oligonucleotides was: 5′-GGAAGUUCCAAGUGGAUAUTT-3′;5′-AUAUCCACUUGGAACUUCCTT-3′. siRNA2–769:5′- GUGGGAACAAGCACAGAAUTT-3′;5′-AUUCUGUG CUUGUUCCCACTT-3′. siRNA3–898:5′-CGCUGACU UUCCUCUUAGUTT-3′;5′-ACUAAGAGGAAAGUCA GCGTT-3′. And negative control siRNA:5′-UUCUCCGAACGUGUCACGUTT-3′;5′-ACGUGACA CGUUCGGAGAATT-3′. The siRNAs targeting CBP were also designed and synthesized by ShangHai GenePharmaCo(Shanghai, China). siRNA1–761:5′-GGCCUCCUCAAUAGUAACUTT-3′;5′-AG UUACUAUUGAGGAGGCCTT-3′. siRNA2–2209:5′-GA GGUCGCGUUUACAUAAATT-3′;5′-UUUAUGUAAAC GCGACCUCTT-3′. And negative control siRNA:5′-UUCUCCGAACGUGUCACGUTT-3′;5′-ACGUGACAC GUUCGGAGAATT-3′.

### Transient transfection

To overexpress CBP in H1299 cells, cells plated in 96-well plates (5,000 cells/well) or six-well plates (200,000 cells/well) were transfected with pcDNA3.1- CBP or control vector plasmids with Liposome particles. To inhibit CBP or RFPL3 expression, the cells were transfected with CBP specific siRNA (10 μmol/L) or RFPL3 specific siRNA and nonspecific siRNA (10 μmol/L). To overexpress RFPL3 in H460 cells, cells plated in six-well plates (200,000 cells/well) were transfected with RFPL3 plasmids or control vector plasmids with Liposome particles. Forty-eight hours after transfection, protein expression and cell viability were tested by Western blot, RT-PCR and MTT analysis, respectively.

### Immunofluorescence assay

The lung cancer cells were seeded onto chamberslides in a 6-well plate and fixed with 4% paraformaldehyde (w/v) for 30 min, washed for 10 min with PBS three times and permeabilized with 0.2% (w/v) Triton X-100 in PBS for 5 min. The blocking step was performed for 30 min in PBS containing 1% bovine serum albumin (BSA). Cells were then incubated overnight with the primary antibodies against RFPL3 and CBP diluted in PBS containing 10% BSA. After PBS washings, cells were incubated for 1 h with secondary antibodies conjugated with fluorescein isothiocyanate or tetra methyl rhodamine isothiocyanate (TRITC) in a moist environment in dark. After several additional washing steps, the coverslips were mounted with Hydromount containing DAPI to stain the nuclei (Beyotime, China). The localization of RFPL3 and CBP protein was examined using a Leica DM 14000B confocal microscopy.

### Treatment of lung cancer cells with CBP inhibitor

To inhibit the histone acetyltransferase (HAT) activity of CBP in lung cancer cells, c646, a competitive HAT inhibitor of CBP (Sigma-Aldrich, SML0002), was used to treat different cells. 48 hours after treatment, cytoplasm and nuclear fractions were isolated, and analyzed as described below.

### Cell viability assay

Cell viability was determined by MTT assay (Roche Diagnosis, Indianapolis, IN) according to the manufacturer's protocol. Briefly, H1299 cells plated in 96- well plates (2000 cells/well) were treated with pcDNA3.1-CBP, or control vector pcDNA3.1-Lac Z, RFPL3 siRNA or control siRNA at the indicated doses. H1299 cell lines stably expressing RFPL3 were also seeded in 96-well plates (2000 cells/well), and treated with CBP siRNA or control siRNA, or pcDNA3.1- CBP or control vector pcDNA3.1-LacZ. 48 hours after treatment, cell viability was determined.

### Promoter activity and dual-luciferase assay

H1299 cells (2 × 10^5^ cells/well) were seeded into six-well plates, cultured overnight, and transfected with the hTERT promoter-luciferase plasmids (1 μg pGL3-hTERT-400 plasmids per well) mediated by DC-nanoparticles. Meanwhile, cells were co-transfected with CBP overexpression vector(pcDNA3.1-CBP), or control vector (pcDNA3.1-LacZ plasmids) and RFPL3 specific siRNA(RFPL3 specific siRNA) or nonspecific siRNA. In addition, the H1299 cell lines stably expressing RFPL3 were co-transfected with CBP specific siRNA or nonspecific siRNA. Transfection efficiency was normalized by co-transfection with Renilla luciferase reporter. Luciferase activity were quantified using a luciferase reporter assay kit(BioVision,http://Inc.CA, USA).

### Telomerase activity assays

Telomerase activity was analyzed by telomerase PCR enzyme-linked immunosorbent assay kit (Roche Applied Science) as described previously [15].

### Streptavidin-agarose pulldown assay

Binding of transcriptional factors or co-activators to hTERT promoter DNA was examined by streptavidin-agarose pulldown assay. A biotin-labeled double-strand DNA probes corresponding to hTERT promoter sequence −378 to +60 was designed and synthesized by Sigma-aldrich (St.Louis, MO) as described previously [15]. Briefly, cells were grown to 80–90% confluence in 150cm^2^ flasks and nuclear extracts were prepared. The binding assay was performed by mixing 400 μg nuclear extract proteins, 10 ug DNA probes, and 100μl streptavidin-agarose beads (Sigma-aldrich, American). The mixture was incubated at room temperature for 2 h with shaking and centrifuged to pull down the DNA-protein complex. The beads were washed by cold PBS three times and the bound proteins were further eluted by being boiled at 95°C for 5 mins for Western blot analysis.

### Human lung adenocarcinoma specimens and immunohistochemistry staining

The human lung adenocarcinoma tissue microarray used for immunostaining analysis of RFPL3 and CBP protein expression was purchased from Shanghai Outdo Biotech (Shanghai, China) and contains 100 lung adenocarcinomas and their corresponding adjacent non-malignant lung tissues. The overall survival (OS) for the corresponding patients was calculated from the day of surgery to the day of death or to the last follow-up. The tissue microarray (TMA) slides were deparaffinized in xylene and rehydrated through graded alcohol. Submerged into EDTA antigenic retrieval buffer and microwaved for antigenic retrieval, followed by 3% hydrogen peroxide in methanol to quench the endogenous peroxidase activity and incubation with 3% bovine serum albumin to block the nonspecific binding. Rabbit polyclonal anti-CBP (1:100; Santa Cruz) and RFPL3 (1:50; abcam) was incubated with the TMA overnight at 4°C. For negative controls, the primary antibody was replaced by normal rabbit serum. After washing, the TMA were treated with biotinylated anti-rabbit secondary antibody (proteintech, US), followed by incubation with streptavidin horseradish peroxidase complex (CST). The degree of immunostaining were reviewed and scored by two independent observers. The proportion of the stained cells and the extent of the staining were used as criteria of evaluation. For each case, at least 1,000 tumor cells were analyzed and the percentage of positively nuclear stained tumor cells was recorded. For each sample, the proportion of RFPL3 and CBP-expressing cells varied from 0% to 100%, and the intensity of nuclear staining varied from weak to strong. One score was given according to the percent of positive cells as: <5% of the cells:1 point; 6–35% of the cells:2 point; 36–70% of the cells:3 point; >70% of the cells: 4 point. Another score was given according to the intensity of staining as negative staining: 1 point; weak staining (light yellow): 2 point; moderate staining (yellowish brown): 3 point; and strong staining (brown): 4 point. A final score was then calculated by averaging the above two scores. If the final score was equal or bigger than four, the tumor was considered high expression; otherwise, the tumor was considered low expression [48].

### Determination of acetylated RFPL3

RFPL3 in nuclear extracts was immunoprecipitated with a specific antibody against RFPL3 or acetylation. After incubation with protein A/G agarose beads, the immunocomplexes were washed extensively, boiled at 95°C for 5mins, separated by SDS-PAGE and analyzed by Western blotting using a pan-Acety antibody or RFPL3 antibody.

### Statistical analysis

Student's *t*-test was used to compare two independent groups of data. ROC curve analysis was utilized to define the cutoff score for high expression of RFPL3 and CBP. Pearson's correlation test was applied to analyze the association between CBP, RFPL3 and hTERT abundance. Survival curves were constructed using the Kaplan-Meier method and were compared using the log-rank test. Statistical analyses were performed using SPSS 17.0 software. Results were shown as mean ± SE. *P* < 0.05 was considered to be statistically significant.
